# VITAL phase 2 study: Upfront 5‐fluorouracil, mitomycin‐C, panitumumab and radiotherapy treatment in nonmetastatic squamous cell carcinomas of the anal canal (GEMCAD 09‐02)

**DOI:** 10.1002/cam4.2722

**Published:** 2019-12-18

**Authors:** Jaime Feliu, Rocio Garcia‐Carbonero, Jaume Capdevila, Inmaculada Guasch, Vicente Alonso‐Orduna, Carlos Lopez, Pilar Garcia‐Alfonso, Carmen Castanon, Isabel Sevilla, Laura Cerezo, Carles Conill, Begona Quintana‐Angel, Maria E. Sanchez, Ismael Ghanem, Marta Martin‐Richard, Miriam Lopez‐Gomez, Ana Leon, Monica Caro, Teresa Fernandez, Joan Maurel

**Affiliations:** ^1^ Department of Medical Oncology CIBERONC Catedra UAM‐AMGEN Hospital Universitario La Paz Madrid Spain; ^2^ Department of Medical Oncology Hospital Universitario Virgen del Rocio Sevilla Spain; ^3^ Department of Medical Oncology imas12 UCM CNIO CIBERONC Hospital Universitario 12 de Octubre Madrid Spain; ^4^ Department of Medical Oncology Hospital Universitari Vall d'Hebron Vall Hebron Institute of Oncology (VHIO) Barcelona Spain; ^5^ Department of Medical Oncology Hospital Althaia‐Manresa Manresa Spain; ^6^ Department of Medical Oncology Instituto de Investigacion Sanitaria de Aragon Hospital Universitario Miguel Servet Zaragoza Spain; ^7^ Department of Medical Oncology Hospital Universitario Marques de Valdecilla Santander Spain; ^8^ Department of Medical Oncology Hospital General Universitario Gregorio Maranon Madrid Spain; ^9^ Department of Medical Oncology Hospital Virgen Blanca Leon Spain; ^10^ Investigacion Clinica y Traslacional en Cancer Instituto de Investigaciones Biomedicas de Malaga (IBIMA) Hospitales Universitarios Regional y Virgen de la Victoria Malaga Spain; ^11^ Department of Radiation Oncology Hospital Universitario de La Princesa Madrid Spain; ^12^ Department of Radiation Oncology Hospital Clinic IDIBAPS University of Barcelona Barcelona Spain; ^13^ Department of Radiation Oncology Hospital Universitario Virgen del Rocio Sevilla Spain; ^14^ Department of Oncology Hospital Universitario La Paz Madrid Spain; ^15^ Department of Medical Oncology Hospital de la Santa Creu i Sant Pau Barcelona Spain; ^16^ Department of Medical Oncology Hospital Universitario Infanta Sofia Madrid Spain; ^17^ Department of Medical Oncology Hospital Universitario Fundacion Jimenez Diaz Madrid Spain; ^18^ Department of Radiation Oncology ICO Badalona Hospital Universitari Germans Trias i Pujol Badalona Spain; ^19^ Department of Medical Oncology Hospital Son Llatzer Palma de Mallorca Spain; ^20^ Department of Medical Oncology Hospital Clinic Translational Genomics and Targeted Therapeutics in Solid Tumours Group IDIBAPS University of Barcelona Barcelona Spain

**Keywords:** chemotherapy, radiotherapy, rectal cancer, target therapy

## Abstract

**Aim:**

VITAL, a phase II single‐arm study, aimed to evaluate efficacy and safety of panitumumab addition to 5‐fluorouracil (5‐FU), mitomycin‐C (MMC) and radiotherapy (RT) in patients with localized squamous cell carcinoma of the anal canal (SCCAC).

**Methods:**

Adult, treatment‐naïve SCCAC patients (Stage T2‐T4, any N, M0) and ECOG‐PS ≤2, received panitumumab (6 mg/kg, day 1 and Q2W; 8 weeks), 5‐FU (1000 mg/m^2^/d, days 1‐4 and 29‐32), MMC (10 mg/m^2^, days 1 and 29) and RT 45 Gy (1.8 Gy/fraction) to the primary tumor and mesorectal, iliac and inguinal lymph nodes, plus 10‐15 Gy boost dose to the primary tumor and affected lymph nodes. The primary objective was disease free survival rate (DFS) at 3‐years (expected 3‐year DFS rate: 73.7 ± 12%).

**Results:**

Fifty‐eight patients (31 women; median age: 59 years; ECOG‐PS 0‐1:98%; TNM II [29%] (T2 or T3/N0/M0)/IIIA (T1‐T3/N1/M0 or T4/N0/M0) [21%]/IIIB (T4/N1/M0 or any T/N2 or N3/M0) [47%]/nonevaluable [4%]) were included. The median follow‐up was 45 months. The 3‐year DFS rate was 61.1% (95% CI: 47.1, 72.4). The 3‐year overall survival rate was 78.4% (95% CI: 65.1, 87.1). Eighteen patients (31.0%) required a colostomy within 2 years posttreatment. Grade 3‐4 toxicities were experienced by 53 (91%) patients. Most common grade 3‐4 treatment‐related events were radiation skin injury (40%) and neutropenia (24%). No toxic deaths occurred. Improved efficacy in colostomy‐free survival and complete response rate was observed in human papilloma virus positive patients.

**Conclusions:**

Panitumumab addition to MMC‐5FU regimen in SCCAC patients increases toxicity and does not improve patients’ outcomes. RT plus MMC‐5FU remains the standard of care for localized SCCAC patients.

## Graphical abstract

VITAL, a phase II single‐arm study, was aimed to evaluate efficacy and safety of panitumumab addition to 5‐fluorouracil (5‐FU), mitomycin‐C (MMC) and radiotherapy in patients with localized squamous cell carcinoma of the anal canal (SCCAC). The study concluded that panitumumab addition to MMC‐5FU regimen in SCCAC patients increases toxicity and does not improve patients’ outcomes.

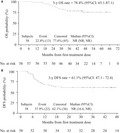

## 1 INTRODUCTION

Anal cancer is a relatively rare cancer which tends to be neglected. However, in 2008 the approximate number of cases was estimated to be 27 000 worldwide.^1^, ^2^ In the United States (US), by 2018, anal cancer or anorectum cancer was expected to account for approximately 2.7% of all digestive cancers.^1^, ^2^

The combination of 5‐fluorouracil (5‐FU), mitomycin‐C (MMC), and radiotherapy (RT) has been established as the standard of care in Europe and the United States since the 1970s.^2^, ^3^ This treatment achieves a 5‐year overall survival (OS) rate of 70%‐80%,^4^, ^5^ and is particularly effective in cT1/T2 tumors achieving long lasting complete tumor regression in 80%‐90% of cases. However, in more advanced anal cancers (T3‐4 or N+), the 3‐year OS rate is 70% and disease‐free survival (DFS) rate is around 50%.^6^ In recent years, several strategies have been studied to improve these results, such as adding induction or consolidation chemotherapy, increasing the RT boost dose or replacing MMC with cisplatin during chemoradiotherapy.^4^, ^5^, ^7^ The substitution of MMC for cisplatin is accepted in the clinic, as similar results have been observed for both treatments with less myelotoxicity.^2^, ^5^ Additionally, capecitabine has been considered as an alternative to infused 5‐FU.^2^, ^3^ However, 5‐FU/MMC + RT remains as the standard of care.

Squamous cell carcinoma of the anal canal (SCCAC), has been associated with many risk factors, most importantly with human papilloma virus (HPV) infection.^8^, ^9^, ^10^, ^11^, ^12^, ^13^, ^14^ The HPV‐associated E5 protein amplifies the mitogenic signals through the epidermal growth factor receptor (EGFR) which is broadly expressed in squamous cell carcinomas such as those from anogenital tract and oropharynx.^15^, ^16^, ^17^ Panitumumab and cetuximab are currently the two monoclonal anti‐EGFR antibodies more widely developed in the clinic. Both anti‐EGFR antibodies are potential treatments for SCCAC patients as not only 80%‐90% of SCCAC tumors express EGFR, but also <5% present *KRAS* mutations, which are associated with lack of activity in monoclonal anti‐EGFR antibodies.^17^

In addition, RT can induce EGFR expression in cancer cells, resulting in acquired resistance. Anti‐EGFR antibodies might help overcome this resistance.^18^ Though there have been previous studies assessing the addition of cetuximab to RT regimens in nonmetastatic SCCAC patients, no studies have been performed to date evaluating the addition of panitumumab.^19^, ^20^, ^21^, ^22^ In addition, previous studies have used chemotherapy regimens based on cisplatin‐5‐FU combinations, although the combination with MMC‐5‐FU is still considered standard by many authors.^23^ This study aimed to evaluate the efficacy and safety of the addition of panitumumab to 5‐FU, MMC and RT standard treatment in patients with SCCAC.

## 2 MATERIAL AND METHODS

Extended methodological details are provided in the Supplementary material. This phase II, open‐label, multicentre, single‐arm trial was conducted in 25 centers in Spain (VITAL Study [GEMCAD‐09‐02], http://clinicaltrials.gov: NCT01285778, EudraCT Number: 2010‐018430‐48). The study was conducted in accordance with the ethical principles of the Declaration of Helsinki and Good Clinical Practice guidelines. The ethics committee at each participating centre and local authorities approved the study protocol and its amendments. All patients provided written informed consent prior to study entry.

### 2.1 Eligibility criteria

Patients were required to have histologically or cytologically confirmed SCACC with T2‐T4 stage and any N stage; age ≥18 years; Eastern Cooperative Oncology Group performance status (ECOG‐PS) 0‐2 and no prior RT or chemotherapy for this malignancy as well as no metastasis.

### 2.2 Study treatments

Patients received treatment with panitumumab (Vectibix^®^; Amgen) 6 mg/kg intravenously (IV) on day 1 and every 2 weeks for 8 weeks. Panitumumab treatment was followed by 5‐FU 1000 mg/m^2^/d by continuous IV infusion on days 1‐4 and 29‐32, and MMC 10 mg/m^2^ IV on days 1 and 29. RT was given on days 1‐37 to a total dose of 45 Gy (1.8 Gy/fraction, 5 fractions per week) to the primary tumor and mesorectal, iliac and inguinal lymph nodes, plus a boost dose of 10‐15 Gy to the primary tumor and affected lymph nodes. Intensity modulated radiation therapy or 3‐D conformal RT was used depending on the center's availability following protocol guidelines (Figure S1).

### 2.3 Study outcomes

The primary outcome measure in this study was DFS rate at 3 years. Secondary outcomes included: complete response (CR) rate, local‐regional failure (LRF) free rate, distant failure free rate, cumulative rate of colostomy, colostomy free survival (CFS), recurrence free survival (RFS), OS and safety profile of this combination. Safety profile included the incidence and severity of adverse events (AE) and significant changes in analytical parameters.

### 2.4 Statistical considerations

A sample size of 58 patients with stage ≥T2N0 was calculated in order to have 80% power to detect a relative increment of 3‐year DFS rate of 10% compared to the US Gastrointestinal Intergroup Radiation Therapy Oncology Group (RTOG) 98‐11 (3‐year DFS rate = 68%), and accounting for a dropout rate of 10%. Therefore, the expected 3‐year DFS for this study was 73.7 ± 12% in order to obtain a relative increase of 10% compared to the RTOG 98‐11.^24^

Detailed information on efficacy outcomes and their reporting are provided in the Supplementary material.

## 3 RESULTS

### 3.1 Patient characteristics

**Figure 1:**
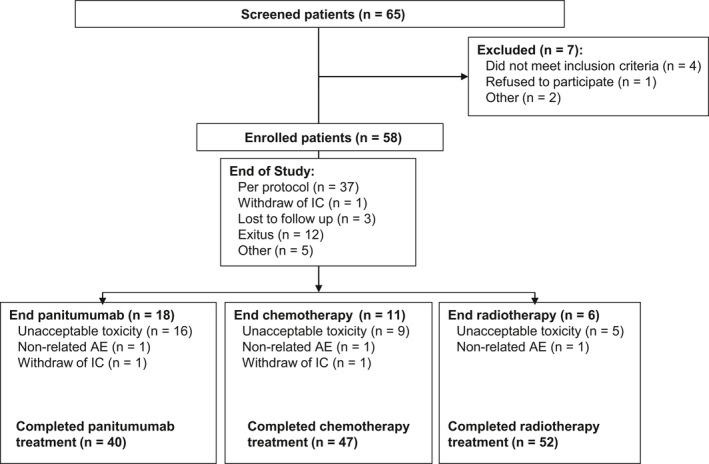
Patient disposition flow chart

A total of 58 patients were enrolled between the January 24, 2011 and the December 2, 2013. All of them were included in the intention‐to‐treat, per‐protocol and safety populations (Figure 1). The overall patient population had a median age of 59.2 (range 33.0‐83.0) years and 53.5% were female (Table 1). A high percentage of tumors was HPV positive (84.8%), whereas only 9% of the patients were human immunodeficiency virus (HIV) positive (percentages calculated over assessed values, tumor samples of 13 patients and 12 patients were not evaluable for HIV and HPV, respectively). Thirty‐six patients (62%) had a primary tumor of ≤5 cm in diameter (T1 or T2) versus 20 (34.5%) who had one of >5 cm (T3 or T4). Thirty‐nine (67%) had positive lymph nodes. The most common TNM tumor stage was IIIB (T4/N1/M0 or any T/N2 or N3/M0) (46.6%) (Table 1).

**Table 1:** Patient characteristics

	Overall population (N = 58)
Median (range) age, year	59.2 (33.0‐83.0)
Sex, female, N (%)	31 (53.5)
Ethnicity, Caucasian, N (%)	57 (98.3)
ECOG performance status, N (%)	
0	24 (41.4)
1	33 (56.9)
2	1 (1.7)
TNM stage, N (%)	
I	0 (‐)
II	17 (29.3)
IIIA	12 (20.7)
IIIB	27 (46.6)
NE	2 (3.5)
HIV positive^a^, N (%)	4 (8.9)
HPV positive^a^, N (%)	39 (84.8)

Abbreviations: ECOG, Eastern Cooperative Oncology Group; HIV, human immunodeficiency virus; HPV, human papillomavirus; NE, not evaluable; TNM, TNM classification of malignant tumors.

a Percentage calculated over observed values (13 patients and 12 patients for the HIV and HPV results respectively were not evaluated).

### 3.2 Treatment administration

Information regarding panitumumab and chemotherapy relative dose intensity, as well as RT administration, are summarized in Table S1. A total of 56 (97%) patients received the two programmed chemotherapy cycles. Most patients received mean ≥90% of the intended dose intensity of panitumumab, MMC and 5‐FU. Dose modifications were required in 9 patients (15.5%) for panitumumab, in 15 patients (25.9%) for 5‐FU and in 11 patients (19.0%) for MMC. Regarding panitumumab: 7 patients (12.1%) had a dose reduction, 17 patients (29.3%) had a dose delay and 16 patients (27.6%) required drug discontinuation. A total of 23 patients received a radiation treatment break due to toxicity with a mean (SD) duration break of 4.9 (4.4) days.

### 3.3 Overall safety

All patients in the study suffered at least one panitumumab‐, chemotherapy‐ and/or RT‐related AE (N = 58). A total of 55 patients (94.8%) developed a grade 3‐4 treatment‐related AE. The most common, occurring in ≥10% of patients, grade 3‐4 treatment‐related AEs are summarized in Table 2. These AEs included: radiation skin injury in 39.7% (31.0% grade 3, 8.6% grade 4), diarrhea in 20.7% (20.7% grade 3, 0% grade 4), neutropenia in 24.1% (19.0% grade 3, 5.2% grade 4), leukopenia in 10.3% (10.3% grade 3, 0% grade 4) and lymphopenia in 15.5% (13.8% grade 3, 1.7% grade 4). A total of 21 (36.2%) patients reported a serious AE (SAE) related to panitumumab, chemotherapy and/or RT. The most common SAEs, occurring in ≥5% of patients included, were: febrile neutropenia in 6.9%, radiation skin injury in 6.9%, diarrhoea in 6.9%, neutropenia in 5.2% and proctalgia in 5.2%. Sixty‐nine percent of patients presented a hematological toxicity (53.4% grade 3‐4) and 77.6% presented diarrhea or colitis (22.4% grade 3‐4) throughout the study. Furthermore, 55.1% of patients presented a skin rash (3.4% grade 3‐4). AEs of special interest are detailed in Table S2.

**Table 2:** Toxicity summary

Treatment related AEs^a^	Safety population (N = 58)
Grade 3‐4 related AEs, N (%)	53 (91.4)
Most common^b^	
Radiation skin injury	23 (39.7)
Neutropenia	14 (24.1)
Diarrhea	12 (20.7)
Lymphopenia	9 (15.5)
Leukopenia	6 (10.3)
Serious treatment‐related AE, N (%)	21 (36.2)
Most common^c^	
Febrile neutropenia	4 (6.9)
Radiation skin injury	4 (6.9)
Diarrhoea	4 (6.9)
Neutropenia	3 (5.2)
Proctalgia	3 (5.2)

Abbreviations: AE, adverse events; RT, radiotherapy; SAE, serious AE.

a AEs related to panitumumab and/or chemotherapy and/or RT.

b Most common (>10%) AE related to any of the three treatments.

c Most common (>5%) SAE related to any of the three treatments.

### 3.4 Primary outcome: 3‐year DFS

**Figure 2:**
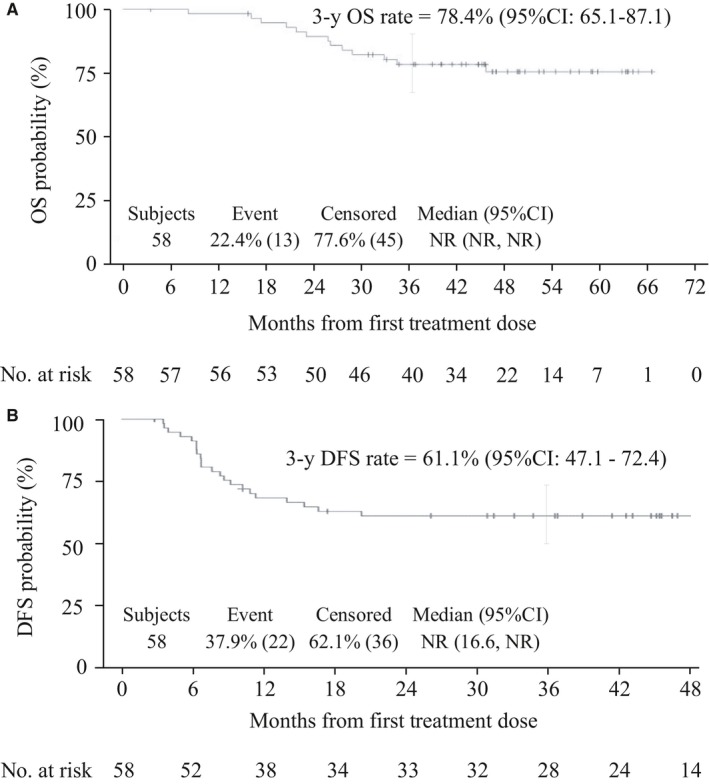
Kaplan‐Meier estimates of disease free survival (A) and overall survival (B)

The median follow‐up time was 45 months (range 3‐67). Treatment efficacy results are summarized in Table 3. The DFS rate at 3 years (95% CI) was 61.1% (47.1, 72.4). Hence, the expected 3‐year DFS rate of 73.7 ± 12% was not achieved (Figure 2).

**Table 3:** Main efficacy results

Percentage (95% CI)	Overall population (N = 58)
DFS rate at 3 y	61.1 (47.1, 72.4)
OS rate at 3 y	78.4 (65.1, 87.1)
CFS rate at 3 y	68.1 (54.2, 78.6)
Clinical CR rate	81.0 (69.8, 92.2)
LRF‐free rate at 3 y	64.8 (50.9, 75.7)
Distant failure free rate, at 3 y	93.0 (82.4, 97.3)
RFS rate at 3 y	75.8 (60.5, 85.8)

Abbreviations: CFS, colostomy‐free survival; CI, confidence interval; CR, complete response; DFS, disease‐free survival, LRF, local‐regional failure, OS, overall survival; RFS, recurrence‐free survival.

### 3.5 Secondary outcome: OS, CR, LRF, distant metastases, and colostomy

The OS at 3 years was 78.4% (95% CI: 65.1, 87.1). The median OS and DFS were not reached. One patient was colostomized prior to his/her study entry. Eighteen patients (31.0%) required a colostomy at some point throughout the study because of anal cancer (N = 17, 94.4%) and poor anorectal function (N = 1, 5.6%). The CFS rate at 3 years was 68.1% (95% CI: 54.2, 78.6).

The CR rate was 81.0% (95% CI: 69.8, 92.2) (N = 47) (Figure S2). Eighteen patients (31.0%) showed only a LRF, two patients (3.4%) showed a local‐regional and distant failure and two patients (3.4%) showed only distant failure. The LRF free and the distant failure free rates at 3 years were 64.8% (95% CI: 50.9, 75.7) and 93.0% (95% CI: 82.4, 97.3), respectively. Taking into account only patients with CR (i.e., 47 patients): 75.8% (95% CI: 60.5, 85.8) of patients were recurrence‐free at 3 years.

### 3.6 Univariate and multivariable analyses

No significant associations were observed between baseline characteristics (age, sex, ECOG status, primary tumor size, HPV, HIV, and cancer stage) or rash severity and the DFS and OS outcomes (Tables S3‐S7).

A statistically significant association was observed between: (a) CFS in HPV negative (N = 7, mean [SD] = 7.6 [0.6]) vs HPV positive (N = 39, 17.3 [0.6]) patients (*P* = .0277), (b) distant failure free rate in HIV negative (N = 41, mean [SD] = 3.6 [‐]) vs HIV positive (N = 4, [‐]) patients (*P* = .0240) and (c) CR rate in HPV positive (N = 39, 84.6% [95% CI 73.3, 95.9]) vs HPV negative (N = 7, 28.6% [95% CI 0.0, 62.1]) patients (*P* < .05) (Figure S3). However, most subpopulations sample sizes were small.

DFS and OS were not associated with any of the prognostic factors studied using a multivariable analysis. All patients were wild type for *RAS* and *EGFR*, so it was not possible to evaluate treatment outcomes according to *RAS* and *EFGR* mutation status.

## 4 DISCUSSION

The results of this study show that panitumumab, when added to MMC‐5FU‐based chemoradiation, does not improve outcomes for the treatment of patients with nonmetastatic SCCAC stage >T1N0. More specifically, the primary objective of the study, which was to demonstrate an improvement in the 3‐year DFS rate, was not achieved.

At the time of the study design, little data on anti‐EGFR agents in SCCAC patients was available. However, current results have shown that several of the initial studies with cetuximab showed high rates of grade 3‐4 radiation‐induced dermatitis and diarrhoea. Toxicity was deemed unacceptable and led to early discontinuation in two of these trials^20^, ^25^ and also showed poor long‐term efficacy results.^21^ Other studies with cetuximab‐chemoradiation did not support further clinical development in phase III trials of cetuximab in patients with SCCAC, either due to small numbers,^26^ excessive toxicity^19^, ^22^ or incomplete/inconclusive efficacy data.^27^, ^28^ It should be noted that those studies testing cetuximab were conducted mainly with cisplatin‐5FU as a chemotherapy partner.^19^, ^20^, ^25^ Our study was the first to investigate the combination of panitumumab with MMC‐5FU‐RT. After our study commenced, the results of a phase I study aiming to determinate the maximum tolerated dose of panitumumab and 5‐FU when combined with MMC‐RT was published. The recommended doses were 3 mg/kg and 400 mg/m^2^, respectively,^29^ considerably lower than the doses used in this study. Also, Aparicio et al communicated the preliminary results of a phase II study that included 45 patients using this scheme. Despite this dose reduction, 89% and 38% of the patients reported a grade 3 or grade 4 toxicity, respectively. With a median follow‐up of 18 months, the RFS and CFS were of 72% and 78%, respectively.^30^

Data from head and neck studies show that toxicity induced by RT associated with cetuximab has a marked inflammatory component, a fact that may have contributed to the poor tolerability of regimens with chemoradiation and cetuximab, associated with unacceptable rates of acute radiation‐induced inflammatory grade 3‐4 AEs. The results of our study would suggest that this tolerability issue is less marked for panitumumab, although the non‐randomized comparison prevents us from drawing any definitive conclusions. In this study, the investigational treatment with panitumumab has resulted in substantial but acceptable and manageable toxicity. Less than 18% of patients presented grade 3‐4 rash. Concerning the most important toxicities such as radiation‐induced epithelitis, diarrhea, and neutropenia, no significant increase in grade 3‐4 AEs compared to what was expected from treatment with MMC‐5FU‐RT was observed.

In the multivariable analysis looking for predictive factors, none of the variables included was associated with DFS or OS. Although no association was found, to the authors' knowledge, this is the first time that the association between skin rash and efficacy has been addressed in patients with SCCAC. This result is not consistent with what has been observed in patients with other solid tumors (i.e., colorectal adenocarcinoma), in whom skin rash severity is associated with improved efficacy of anti‐EGFR agents.^31^ In the multivariable analysis of CR (81% for the whole population), no association with skin rash severity was observed either. However, a remarkable result was found concerning the HPV status. Improved efficacy was observed both in terms of CFS and CR rate in patients with HPV‐positive tumors. Due to the design of the study, it is not possible to determine whether this was a predictive or prognostic factor of panitumumab‐MMC‐5FU‐RT efficacy. However, these results are in line with those observed in patients with squamous cell carcinoma of the oropharynx who have a better prognosis if they are HPV‐positive. In these cases, the possibility of therapeutic de‐escalation has been suggested.^32^ The extrapolation of this concept to patients with HPV‐positive SCCAC is still unknown and could only be confirmed in further larger studies. However, two recent randomized trials have studied this strategy in HPV‐positive patients with oropharyngeal cancer, concluding that, besides cetuximab resulting in lesser toxicities when administered with RT compared with cisplatin + RT, it was found to be inferior in OS.^33^, ^34^ These results suggest that de‐escalation strategy should be carefully used in clinical practice, and also that anti‐EGFR antibodies may not be the optimal therapies for this strategy.

The reason behind the lack of incremental efficacy when anti‐EGFR is added to chemo‐radiotherapy in SCCAC patients remains unknown. Classically, the *RAS* and *EGFR* mutations have been appointed as factors of resistance to anti‐EFGR therapies, but in our study no patient presented with these mutations.

It could be the case that EGFR downstream mutations (ie, activating in PIK3CA, inactivating in PTEN) might mediate resistance to EGFR‐targeted therapies, being more frequent in HPV‐positive oropharyngeal carcinoma.^35^ Actually, some retrospective studies in head and neck tumors suggest that anti‐EGFR treatments might be more efficacious in VPH‐negative tumors.^36^ Additionally, it has been described that FBXW7 mutations are associated with anti‐EGFR resistance.^37^ Finally, it is noteworthy that, besides initial studies suggest an 80%‐90% EGFR overexpression rate in SCCAC tumors,^16^, ^17^ a recent meta‐analysis suggests that this may occur in only 60%, which may be associated with a lower sensitivity to anti‐EGFR therapies.^38^

The two monoclonal antibodies (mAb) cetuximab and panitumumab are often considered equally effective and their selection for clinical use is often a matter of personal choice in colorectal cancer. Yet they are different. Cetuximab is an immunoglobulin G1 isotype mAb, which can elicit immune functions such as antibody‐dependent cell‐mediated cytotoxicity involving natural killer cells, T‐cell recruitment, and T‐cell priming. There is evidence that this process can lead to immunosuppressive feedback loops,^39^ and some have therefore suggested combining checkpoint inhibitors with cetuximab or cobimetinib in microsatellite stable colorectal cancer. Panitumumab, an IgG2 isotype mAb, is not reported to exert these immune effects.

Therapies targeting PD‐1/L1 have produced response rates of 15%‐20% in patients with HPV associated cancers including cervical, anal or head and neck squamous cell carcinoma. Another potential target for these diseases is transforming growth factor‐β (TGF‐β) as genome wide association studies in HPV‐positive cancers have shown TGF‐β to be significantly overexpressed.^40^

The nonrandomized design is the main limitation of this study as it can be associated with a selection bias. It is worth mentioning that male sex and advanced stage have been shown to be poor prognostic markers for LRF and OS.^3^, ^4^ In our study, we included a percentage of men (46%) higher than those found in previous studies which are close to 25%‐30%,^7^, ^19^, ^24^, ^25^, ^26^ and a higher percentage of patients with positives nodes (67%) and with stage IIIB (47%) than those found in previous studies which are close to 30%‐35%.^4^, ^5^ Therefore, our population may be biased toward poor prognosis. However, the phase II design was mandatory as this study is the first conducted and reported, assessing the efficacy and toxicity of panitumumab associated with MMC‐5FU‐based chemoradiation in patients with SCCAC.

It is necessary to continue investigating with new RT techniques and new pharmacological strategies such as immunotherapy with anti‐PD‐1 mAbs,^41^ therapeutic vaccines targeting the E6 and E7 oncoproteins,^42^ PIK3CA mutant targeted therapies (present in 15%‐20% SCCAC tumors), or drugs targeting TGF‐β.^40^

## 5 CONCLUSIONS

The addition of panitumumab to the standard chemoradiation regimen with MMC‐5FU in patients with SCCAC did not result in improved outcomes.

## DATA AVAILABILITY STATEMENT

The data that support the findings of this study are available on request from the corresponding author. The data are not publicly available due to privacy or ethical restrictions.
