# Supplementary material for: VITAL phase 2 study: Upfront 5‐fluorouracil, mitomycin‐C, panitumumab and radiotherapy treatment in nonmetastatic squamous cell carcinomas of the anal canal (GEMCAD 09‐02)
Source: Cancer Med. 2019 Dec 18;9(3):1008–16. doi: 10.1002/cam4.2722 (PMC6997048; doi:10.1002/cam4.2722)
Supplement: Supplementary file 1 [file CAM4-9-1008-s001.docx]

## Supplementary Material

[Supplementary methods 2](#_Toc18658872)

[Study Design 2](#_Toc18658873)

[Eligibility Criteria 2](#_Toc18658874)

[Study treatments 2](#_Toc18658875)

[Follow-up 3](#_Toc18658876)

[Study Objectives 3](#_Toc18658877)

[Study End Point Definitions 3](#_Toc18658878)

[Statistical Considerations 4](#_Toc18658879)

[Supplementary Tables and Figures 5](#_Toc18658880)

[Supplementary Tables 5](#_Toc18658881)

[Table S1 Treatment exposure 5](#_Toc18658882)

[Table S2 Grade 1-4 adverse events of special interest 6](#_Toc18658883)

[Table S3 DFS associations with baseline characteristics: univariate analysis with Log-rank test. 7](#_Toc18658884)

[Table S4 OS associations with baseline characteristics: univariate analysis with Log-rank test. 8](#_Toc18658885)

[Table S5 CFS associations with baseline characteristics: univariate analysis with Log-rank test. 9](#_Toc18658886)

[Table S6 DFFS associations with baseline characteristics: univariate analysis with Log-rank test. 10](#_Toc18658887)

[Table S7 Complete response rate associated with baseline characteristics. 11](#_Toc18658888)

[Supplementary Figures 12](#_Toc18658889)

[Figure S1 Treatment Schedule 12](#_Toc18658890)

[Figure S2 Patient treatment response flow chart 13](#_Toc18658891)

[Figure S3 Univariate analysis of CR rate association with baseline characteristics/rash severity 14](#_Toc18658892)

[References 15](#_Toc18658893)

## Supplementary methods

### Study Design

This phase II, open-label, multicentre, single-arm trial was conducted in 25 centres in Spain (VITAL Study [GEMCAD-09-02], EudraCT Number: 2010-018430-48, NCT01285778). The study was conducted in accordance with the ethical principles of the Declaration of Helsinki and Good Clinical Practice guidelines. The ethics committee at each participating centre and local authorities approved the study protocol and its amendments. All patients provided written informed consent prior to study entry.

### Eligibility Criteria

Inclusion criteria were: able to provide written informed consent; aged ≥ 18 years old; histologically or cytologically confirmed SCAC; radiologically determined T2-T4 stages and any N stage (pelvic or inguinal) determined by MRI; an ECOG performance status of 0-2; in the seven days prior to the start of study treatment: adequate haematology (neutrophils ≥ 1.5 x 10^9^ cells / L; platelets ≥ 100 x 10^9^ cells/ L; haemoglobin ≥ 9 g / dL), liver function (total bilirubin count ≤ 1.5 x upper limit of normal [ULN], ALT and AST ≤ 2.5 x ULN), and renal function (creatinine clearance ≥ 50 mL / min). Exclusion criteria were: metastatic disease; HIV infection (except for patients with an undetectable viral load and CD4 cell count > 400 / mL who are eligible to participate in the study); past or current history (within 5 years prior to initiation of treatment) of other malignancies except metastatic colorectal cancer (patients with non-melanoma skin cancer treated with curative intent or in situ carcinoma of the cervix were eligible); clinically significant cardiovascular disease, such as myocardial infarction (<6 months prior to initiation of therapy), unstable angina, congestive heart failure, arrhythmia requiring medication, or uncontrolled hypertension; treatment with any other product under investigation or participation in another clinical study within 30 days prior to inclusion in this study; known hypersensitivity to any of the study drugs or their excipients; indications of another disease, metabolic dysfunction, physical examination or analytical finding that may be indicative of a disease or disorder for which the use of an investigational drug is contraindicated or which poses a high risk of complications related to the disease treatment for the patient; and men and women of childbearing age who did not wish to use effective contraceptive methods.

### Study treatments

Patients received treatment with panitumumab (Vectibix^®^, Amgen) 6 mg/kg intravenously (IV) on day 1, and every 2 weeks for 8 weeks. Panitumumab treatment was followed by 5-FU 1,000 mg/ day by continuous IV infusion on days 1-4 and 29-32 and mitomycin-C 10 mg / m^2^ IV on days 1 and 29. Mitomycin-C was administered with standard prophylactic premedication 30 minutes prior to the administration. Panitumumab and chemotherapy were administered together with radiotherapy treatment which was administered on day 1-37 to a total dose of 45 Gy (1.8 Gy per fraction, 5 fractions per week), to the regional and inguinal ganglia and the primary tumour, and followed by a boost of 10-15 Gy in the primary tumour and affected lymph nodes. Intensity modulated radiation therapy or 3-D conformal radiotherapy was used depending on the centre’s availability following guidelines outlined in this protocol (Supplementary Figure S1) [1]. Panitumumab administration was suspended if a skin- or nail-related serious adverse event (AE) occurred, in the case of infusion reactions or grade 3-4 toxicity, or in case of symptomatic hypomagnesaemia and/or hypocalcaemia that persists despite the intensive replacement of calcium and/or magnesium, grade 3-4 nausea, vomiting or diarrhoea that persist despite best supportive care, or grade ≥3 anaemia or grade 4 thrombocytopenia that persist despite blood transfusions or cytokine therapy.

Panitumumab was restarted once AEs related toxicity improved to grade ≤2 or basal level, the patient skin or the nails toxicity has recovered to a tolerable symptomatic state, systemic steroids are no longer required and antibiotic or antifungal treatments are not necessary.

Mitomycin-C and 5-FU were suspended if Grade 2 neutropenia, thrombocytopenia or diarrhoea were observed (NCI CTC-AE Version 3.0). Both treatments were restarted within 2 weeks of suspension if: an absolute neutrophil count (ANC) >1.5 x 10^9^/L, or a total platelet count >100 x 10^9^/L were reached, or asthenia improved to grade ≤2 or the rest of AE related toxicities improved to grade ≤1. Both treatments were reduced (50% of prescribed dose) if Grade 3-4 neutropenia/neutropenic fever, thrombocytopenia, diarrhoea were observed. Both treatments were discontinued after 3 (neutropenia) or 4 (thrombocytopenia or diarrhoea) weeks if not resolved. Mitomycin-C was discontinued if haemolytic uremic syndrome or pulmonary fibrosis were observed.

### Follow-up

Once the study treatment was completed or discontinued, a safety follow-up visit was performed 30-33 days after the last treatment dose administration. In addition, in those patients with no disease progression at the end of the treatment (including patients who discontinued the study treatment for any reason, for instance due to unacceptable toxicities) were followed until disease progression or end of the study. These long-term follow-up safety visits were performed every 3 ± 1 months within the first follow-up year from 60 ± 7 days after the last radiotherapy dose and every 6 ± 1 months from the first follow-up year onwards.

### Study Objectives

The primary objective of this study was DFS rate at three years. Secondary objectives included: complete response (CR) rate, locoregional failure (LRF) free rate, distant failure free rate, cumulative rate of colostomy, colostomy free survival (CFS), recurrence free survival (RFS) overall survival (OS), and safety profile of this combination. Safety profile included the incidence and severity of AEs and significant changes in analytical parameters. The evaluation of treatment outcomes according to *RAS* and *EFGR* mutation status was planned as an exploratory objective

### Study End Point Definitions

Disease free survival was defined as number of months between the first treatment dose until the first treatment failure (defined as disease progression by MRI or CT, persistence of disease confirmed by biopsy performed at least 6 months after end of treatment, rescue surgery/colostomy by progression or death by progression). Progression free survival was defined as the number of months between the first treatment dose until progression, rescue surgery/colostomy due to progression or death. Overall survival was defined as number of month between first treatment dose and death for any reason. LRF was defined as relapse of disease in the anal canal and/or regional organs and/or regional lymph nodes. All other relapses were considered distant failures. Colostomy free survival rate was defined as the number of patients alive and without a colostomy.

Response was evaluated clinically or radiologically according to Response Evaluation Criteria in Solid Tumours (RECIST) criteria (version 1.1). Tumour assessments were made by physical examination and MRI or TC. Recurrence free survival was defined as number of months between the first CR to the treatment until the first treatment failure (disease progression analysed by MRI or TC, rescue surgery/colostomy due to progression or death due to progression).

Severity of AEs was categorised in accordance with Institute Common Adverse Events Criteria, version 3.0.

### Statistical Considerations

A sample size of 58 patients with stage >T2N0 was calculated in order to have 80% power to detect a relative increment of 3-year DFS rate of 10% compared to the US Gastrointestinal Intergroup Radiation Therapy Oncology Group (RTOG) 98-11 (3-year DFS rate = 68%), and accounting for a dropout rate of 10%. Therefore, the expected 3-year DFS for this study was 73.7% ± 12% in order to obtain a relative increase of 10% compared to the RTOG 98-11 [2].

All efficacy variables were reported using descriptive statistics that include point estimates and time-to-event variables were estimated by the Kaplan-Meier (KM) method both with their respective 95% confidence intervals.

The analysis set included all patients who received at least one dose of panitumumab (intention-to-treat population). Adverse events were reported using frequency counts and percentages; analytical values using descriptive statistics. The safety set included all patients who signed the informed consent form and received at least one dose of panitumumab or chemotherapy.

An intermediate analysis was conducted with efficacy and safety data from a cut-off date of 3 October 2013. This intermediate analysis has been previously reported at the 2014 American Society of Clinical Oncology (ASCO) annual meeting, May 30 – June 3, Chicago, USA.

Data analysis was performed using the SAS® statistical package for Windows (Version 7.11 SAS Institute Inc., Cary, USA).

## Supplementary Tables and Figures

## Supplementary Tables

### Table S1 Treatment exposure

| Treatment | **Overall population (N=58)** |
| --- | --- |
| Panitumumab (intended dose intensity), % |  |
| Mean (SD)  Median (range) | 90 (17)  99 (38 – 106) |
|  |  |
| Mitomycin C (intended dose intensity), % |  |
| Mean (SD)  Median (range) | 93 (13)  100 (44 – 102) |
|  |  |
| 5-fluorouracil (intended dose intensity), % |  |
| Mean (SD)  Median (range) | 91 (14)  100 (44 – 102) |
|  |  |
| Radiation (cumulative dose), Gy* |  |
| Mean (SD)  Median (range) | 60.0 (22.3)  59.4 (36, 209) |
| Abbreviations: SD, standard deviation  * N=55, two patients presented no data and one patient presented erroneous data collection or entry error therefore three patients were not included in the analysis. | |

### Table S2 Grade 1-4 adverse events of special interest

|  |  | **Worst Grade** | | |  |
| --- | --- | --- | --- | --- | --- |
|  | No AE | 1 | 2 | 3 | 4 |
| Type of AE, n (%) |  |  |  |  |  |
| Haematological toxicity* | 17 (29.3) | 2 (3.5) | 8 (13.8) | 23 (39.7) | 8 (13.8) |
| Skin Rash^†^ | 7 (12.1) | 20 (34.5) | 21 (36.2) | 8 (13.8) | 2 (3.5) |
| Diarrhoea/Colitis | 13 (22.4) | 13 (22.4) | 19 (32.8) | 12 (20.7) | 1 (1.7) |
| Abbreviations: AE=Adverse Event  *AE under the SOC “Blood and lymphatic system disorders” as well as the preferred terms: International normalised ratio, haemoglobin decreased and platelet count decreased  ^†^AEs under the following preferred terms: acne, dermatitis, dermatitis acneiform, solar dermatitis, erythema, rash and toxic skin eruption | | | | | |

### Table S3 DFS associations with baseline characteristics: univariate analysis with Log-rank test.

| **Baseline Characteristics** | **DFS**  mean (SD), months | **P-value** |
| --- | --- | --- |
| Age Years |  |  |
| <65 (n=41) | 14.75 (1.08) |  |
| ≥65 (n=17) | 15.60 (0.75) | 0.0988 |
| Sex |  |  |
| Male (n=27) | 16.12 (1.27) |  |
| Female (n=31) | 12.69 (0.77) | 0.8591 |
| ECOG |  |  |
| 0 (n=24) | 15.42 (1.37) |  |
| 1 (n=33) | 12.96 (0.76) |  |
| 2 (n=1) | 16.62 () | 0.6235 |
| Primary tumour size by MR |  |  |
| ≤50mm (n=38) | 15.63 (1.01) |  |
| >50mm (n=20) | 12.82 (1.12) | 0.4784 |
| Basal HPV* |  |  |
| Positive (n=39) | 14.50 (0.63) |  |
| Negative (n=7) | 6.03 (0.45) | 0.1360 |
| Basal HIV* |  |  |
| Positive (n=4) | 8.51 (1.82) |  |
| Negative (n=41) | 16.51 (0.98) | 0.1299 |
| Rash NCI-CTC Grade |  |  |
| No Rash (n=7) | 17.25 (1.65) |  |
| Mild-moderate (n=41) | 13.43 (0.79) |  |
| Severe-life threatening (n=10) | 7.91 (0.26) | 0.7997 |
| TNM Stage |  |  |
| Stage II (n=17) | 11.92 (1.09) |  |
| Stage III (n=39) | 10.39 (0.60) | 0.7478 |
| Abbreviations: CR = complete response, DFS = disease free survival, ECOG = Eastern Cooperative Oncology Group, HIV = Human Immunodeficiency Virus, HPV = Human papillomavirus, NCI-CTC = National Cancer institute - common toxicity criteria  *Note, not evaluable patients in HPV (n=12) and HIV (n=13)  **Mean [Standard Deviation] of disease free survival variable is reported, as the estimated median survival was not reached in this study.** | | |

### Table S4 OS associations with baseline characteristics: univariate analysis with Log-rank test.

| **Baseline Characteristics** | **OS** | **P-value** |
| --- | --- | --- |
| Age Years |  |  |
| <65 (n=41) | 41.30 (1.58) |  |
| ≥65 (n=17) | 25.10 (0.67) | 0.8833 |
| Sex |  |  |
| Male (n=27) | 31.89 (1.06) |  |
| Female (n=31) | 42.06 (1.94) | 0.2129 |
| ECOG |  |  |
| 0 (n=24) | 41.80 (1.74) |  |
| 1 (n=33) | 26.99 (0.89) |  |
| 2 (n=1) | ()** | 0.8598 |
| Primary tumour size by MR |  |  |
| ≤50mm (n=38) | 41.91 (1.40) |  |
| >50mm (n=20) | 29.55 (1.77) | 0.7242 |
| Basal HPV* |  |  |
| Positive (n=39) | 41.75 (1.60) |  |
| Negative (n=7) | 32.89 () | 0.1951 |
| Basal HIV* |  |  |
| Positive (n=4) | 33.09 (1.72) |  |
| Negative (n=41) | 40.08 (1.78) | 0.2281 |
| Rash NCI-CTC Grade |  |  |
| No Rash (n=7) | 25.49 (1.69) |  |
| Mild-moderate (n=41) | 32.64 (0.93) |  |
| Severe-life threatening (n=10) | 40.81 (3.73) | 0.2374 |
| TNM Stage |  |  |
| Stage II (n=17) | 33.52 (0.79) |  |
| Stage III (n=39) | 40.01 (1.83) | 0.5493 |
| Abbreviations: CR = complete response, ECOG = Eastern Cooperative Oncology Group, HIV = Human Immunodeficiency Virus, HPV = Human papillomavirus, NCI-CTC = National Cancer institute - common toxicity criteria, OS = overall survival  *Note, not evaluable patients in HPV (n=12) and HIV (n=13)  ** All patients were still alive by 54 months  **Mean [Standard Deviation] of overall survival variable is reported as the estimated median survival was not reached in this study.** | | |

### Table S5 CFS associations with baseline characteristics: univariate analysis with Log-rank test.

| **Baseline Characteristics** | **CFS** | **P-value** |
| --- | --- | --- |
| Age Years |  |  |
| <65 (n=41) | 18.55 (1.12) |  |
| ≥65 (n=17) | 17.30 (1.15) | 0.1555 |
| Sex |  |  |
| Male (n=27) | 18.95 (1.34) |  |
| Female (n=31) | 14.92 (0.77) | 0.4576 |
| ECOG |  |  |
| 0 (n=24) | 19.72 (1.27) |  |
| 1 (n=33) | 14.41 (0.83) |  |
| 2 (n=1) | 18.69 () | 0.5319 |
| Primary tumour size by MR |  |  |
| ≤50mm (n=38) | 19.47 (1.02) |  |
| >50mm (n=20) | 7.77 (0.29) | 0.6227 |
| **Basal HPV*** |  |  |
| **Positive (n=39)** | **17.25 (0.60)** |  |
| **Negative (n=7)** | **7.64 (0.56)** | **0.0277** |
| Basal HIV* |  |  |
| Positive (n=4) | 12.76 (2.14) |  |
| Negative (n=41) | 19.32 (1.10) | 0.1311 |
| Rash NCI-CTC Grade |  |  |
| No Rash (n=7) | 23.29 () |  |
| Mild-moderate (n=41) | 15.55 (0.84) |  |
| Severe-life threatening (n=10) | 12.06 (0.48) | 0.5338 |
| TNM Stage |  |  |
| Stage II (n=17) | 20.83 (1.35) |  |
| Stage III (n=39) | 14.21 (0.72) | 0.6867 |
| Abbreviations: CFS = colostomy free survival, CR = complete response, ECOG = Eastern Cooperative Oncology Group, HIV = Human Immunodeficiency Virus, HPV = Human papillomavirus, NCI-CTC = National Cancer institute - common toxicity criteria  *Note, not evaluable patients in HPV (n=12) and HIV (n=13)  **Mean [Standard Deviation] of colostomy free survival variable is reported as the estimated median survival was not reached in this study.** | | |

### Table S6 DFFS associations with baseline characteristics: univariate analysis with Log-rank test.

| **Baseline Characteristics** | **DFFS** | **P-value** |
| --- | --- | --- |
| Age Years |  |  |
| <65 (n=41) | 13.45 (0.43) |  |
| ≥65 (n=17) | 10.25 () | 0.8214 |
| Sex |  |  |
| Male (n=27) | 3.94 () |  |
| Female (n=31) | 13.49 (0.44) | 0.3623 |
| ECOG |  |  |
| 0 (n=24) | 3.94 () |  |
| 1 (n=33) | 13.52 (0.41) |  |
| 2 (n=1) | ()** | 0.7339 |
| Primary tumour size by MR |  |  |
| ≤50mm (n=38) | 13.86 (0.14) |  |
| >50mm (n=20) | 3.92 (0.03) | 0.4924 |
| Basal HPV* |  |  |
| Positive (n=39) | 13.87 (0.14) |  |
| Negative (n=7) | 3.94 () | 0.6569 |
| **Basal HIV*** |  |  |
| **Positive (n=4)** | **()**** |  |
| **Negative (n=41)** | **3.55 ()** | **0.0240** |
| Rash NCI-CTC Grade |  |  |
| No Rash (n=7) | 13.43 (0.69) |  |
| Mild-moderate (n=41) | 3.93 (0.01) |  |
| Severe-life threatening (n=10) | ()** | 0.0590 |
| TNM Stage |  |  |
| Stage II (n=17) | ()** |  |
| Stage III (n=39) | 13.34 (0.43) | 0.1912 |
| Abbreviations: CR = complete response, DFFS = distant failure free survival, ECOG = Eastern Cooperative Oncology Group, HIV = Human Immunodeficiency Virus, HPV = Human papillomavirus, NCI-CTC = National Cancer institute - common toxicity criteria  *Note, not evaluable patients in HPV (n=12) and HIV (n=13)  ** All patients were still alive by 54 months  **Mean [Standard Deviation] of distant failure free survival variable is reported as the estimated median survival was not reached in this study.** | | |

### Table S7 Complete response rate associated with baseline characteristics.

| **Baseline Characteristics** | **Patients without CR** | **Patients with CR** |
| --- | --- | --- |
| Age Years, n (%[95CI%]) |  |  |
| <65 | 14 (34.15 [19.63, 48.66] ) | 27 (65.85 [51.34, 80.37]) |
| ≥65 | 2 (11.76 [0.00, 27.08]) | 15 (88.24 [72.92, 100.00]) |
| Sex, n (%[95CI%]) |  |  |
| Male | 9 (33.33 [15.55, 51.11]) | 18 (66.67[48.89, 84.45]) |
| Female | 7 (22.58 [7.86, 37.30]) | 24 (77.42 [62.70, 92.14]) |
| ECOG, n (%) |  |  |
| 0 | 7 (29.17 [0.0, 62.8]) | 17 (70.83% [52.6, 89.0]) |
| 1 | 9 (27.27 [9.5, 45.1]) | 24 (72.73 [57.5, 87.9]) |
| 2 | 0 (0.00 [-]) | 1 (100.00 [100.0, 100.0]) |
| Primary tumour size by MR, n (%[95CI%]) |  |  |
| ≤50mm | 9 (23.68 [10.17, 37.20]) | 29 (76.32 [62.80, 89.83]) |
| >50mm | 7 (35.00 [14.10, 55.90]) | 13 (65.00[44.10, 85.90]) |
| Basal HPV, n (%[95CI%])* |  |  |
| Positive | 6 (15.38 [4.06, 26.71]) | 33 (84.62 [73.29, 95.94]) |
| Negative | 5 (71.43 [37.96, 100.00]) | 2 (28.57 [0.00, 62.04]) |
| **Basal HIV, n (%[95CI%])*** |  |  |
| **Positive** | **1 (25.00 [0.00, 67.43])** | **3 (75.00 [32.57, 100.00])** |
| **Negative** | **13 (31.71 [17.46, 45.95])** | **28 (68.29 [54.05, 82.54])** |
| Rash NCI-CTC Grade, n (%[95CI%]) |  |  |
| No Rash | 1 (14.29 [0.00, 40.21]) | 6 (85.71 [59.79, 100.00]) |
| Mild-moderate | 12 (29.27 [15.34, 43.20]) | 29 (70.73 [56.80, 84.66]) |
| Severe-life threatening | 3 (30.00 [1.60, 58.40]) | 7 (70.00 [41.60, 98.40]) |
| TNM Stage, n (%[95CI%]) |  |  |
| Stage II | 4 (23.53 [3.37, 43.69]) | 13 (76.47 [56.31, 96.63]) |
| Stage III | 11 (28.21 [14.08, 42.33]) | 28 (71.79 [57.67, 85.92]) |
| Abbreviations: CR = complete response, ECOG = Eastern Cooperative Oncology Group, HIV = Human Immunodeficiency Virus, HPV = Human papillomavirus, NCI-CTC = National Cancer institute - common toxicity criteria, NE = not evaluated,  *Note, not evaluable patients in HPV (n=12) and HIV (n=13) | | |

## Supplementary Figures

### Figure S1 Treatment Schedule


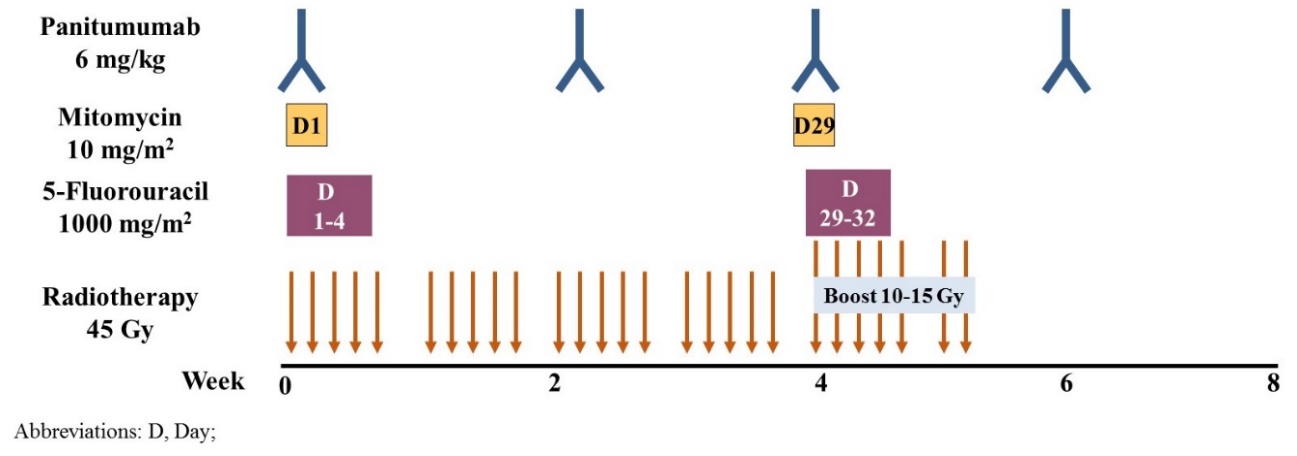


### Figure S2 Patient treatment response flow chart

*Patients which showed CR evaluated radiologically and/or clinically.

$ These patients did not have documented disease progression before end of study. Abbreviations: CR, complete response.

### Figure S3 Univariate analysis of CR rate association with baseline characteristics/rash severity

## References

[1] James RD, Glynne-Jones R, Meadows HM, et al: Mitomycin or cisplatin chemoradiation with or without maintenance chemotherapy for treatment of squamous-cell carcinoma of the anus (ACT II): a randomised, phase 3, open-label, 2 × 2 factorial trial. Lancet Oncol 14:516–524, 2013

[2] Gunderson LL, Winter KA, Ajani JA, et al: Long-term update of US GI intergroup RTOG 98-11 phase III trial for anal carcinoma: survival, relapse, and colostomy failure with concurrent chemoradiation involving fluorouracil/mitomycin versus fluorouracil/cisplatin. J Clin Oncol Off J Am Soc Clin Oncol 30:4344–4351, 2012
